# Type 1 Diabetes Candidate Genes Linked to Pancreatic Islet Cell Inflammation and Beta-Cell Apoptosis

**DOI:** 10.3390/genes8020072

**Published:** 2017-02-16

**Authors:** Joachim Størling, Flemming Pociot

**Affiliations:** 1 Copenhagen Diabetes Research Center (CPH-DIRECT), Department of Pediatrics, University Hospital Herlev and Gentofte, Herlev 2730, Denmark; joachim.stoerling.01@regionh.dk; 2 Department of Clinical Medicine, Faculty of Health and Medical Sciences, University of Copenhagen, Copenhagen 2200, Denmark

**Keywords:** GWAS, beta-cell, gene expression, apoptosis

## Abstract

Type 1 diabetes (T1D) is a chronic immune-mediated disease resulting from the selective destruction of the insulin-producing pancreatic islet β-cells. Susceptibility to the disease is the result of complex interactions between environmental and genetic risk factors. Genome-wide association studies (GWAS) have identified more than 50 genetic regions that affect the risk of developing T1D. Most of these susceptibility loci, however, harbor several genes, and the causal variant(s) and gene(s) for most of the loci remain to be established. A significant part of the genes located in the T1D susceptibility loci are expressed in human islets and β cells and mounting evidence suggests that some of these genes modulate the β-cell response to the immune system and viral infection and regulate apoptotic β-cell death. Here, we discuss the current status of T1D susceptibility loci and candidate genes with focus on pancreatic islet cell inflammation and β-cell apoptosis.

## 1. Introduction

Through the remarkable progress in human genetics, dozens of novel risk alleles that contribute to the likelihood of developing type 1 diabetes (T1D) have been identified over the past decade [1]. Unfortunately, for most of them, the molecular mechanisms of action remain undefined. However, it has become clear that many of the genes associated with the risk-conferring single-nucleotide polymorphisms (SNPs) are expressed in pancreatic β-cells [2,3], raising the exciting possibility that a significant fraction of T1D risk is determined by the β-cell transcriptome controlling the function, survival, and interaction of β-cells with the immune system.

Genetic fine-mapping, genotype–phenotype correlation studies, as well as functional experiments have been employed to identify causal variants and genes acting at the β-cell level. Also, various network and systems biology approaches have been applied to understand the underlying mechanisms of β-cell destruction in T1D. These analyses have proven valuable for prioritizing disease-associated genes, and for providing insight into the possible mechanisms by pathway analyses [4]. Loss- and gain-of-function studies in β-cell model systems have already provided important knowledge of the function of a number of T1D candidate genes and their pathways. These observations suggest that a few major genetically-regulated pathways contribute to β-cell dysfunction and death in T1D, e.g., innate immunity and antiviral activity, and pathways related to β-cell phenotype and susceptibility to pro-apoptotic stimuli [5,6].

The identification of specific genes and the underlying mechanisms involved in β-cell destruction is essential for the development of new therapeutic strategies to improve β-cell function in recent-onset T1D patients and to avoid β-cell impairment in risk individuals.

Most of the disease-associated variants have small individual effects on disease risk and are therefore insufficient to predict disease development or provide immediate diagnostic benefit [7,8]. Thus, functional analysis of risk variants is the most promising approach to determine the exact significances of the associated variation in disease pathogenesis and to define their translational potential.

Interestingly, more than 75% of disease-associated genome-wide association study (GWAS) SNPs map to functional regulatory elements outside protein-coding sequences according to the Encyclopedia of DNA Elements (ENCODE) project [9,10]. Thus, it appears that genetic variants which modulate gene expression, rather than protein sequence, constitute the primary basis of genetic predisposition to disease.

## 2. T1D Pathogenesis

T1D is caused by an immune-mediated targeted destruction of the pancreatic β-cells leading to absolute insulin deficiency. The pathogenesis can be divided into two main phases—the initiation phase involving innate immune mechanisms, and the execution phase involving adaptive immune mechanisms. During the initiation phase, initial β-cell damage and death are induced, leading to the invasion of the islets of activated T-lymphocytes that mediate the killing of the β-cells [11,12,13,14]. Evidence from animal models supports that the Fas/FasL system, perforin/granzyme pathway, and pro-inflammatory cytokines, including interleukin (IL)-1β, interferon (IFN)-γ, and tumor necrosis factor (TNF)-α, contribute to the killing of the β-cells [12,13,15,16].

The complex etiology of T1D is underlined by the fact that the timeframe from initial β-cell damage to manifestation of clinical diabetes may stretch over several years. Studies now suggest that, in most cases, T1D patients have substantial residual β-cell mass at the time of diagnosis. Further, some patients with long-standing T1D still have the capability to produce and secrete low amounts of insulin [17,18], while others, despite having insulin-positive cells in the pancreas, do not secrete detectable C-peptide levels [11,19]. However, a recent report demonstrated that isolated islets from pancreas biopsies from T1D patients at the time of disease onset can regain their insulin secretion following in vitro culture [20]. These findings strongly suggest that the loss of β-cell function in T1D is not solely caused by a decline in β-cell mass, but also caused by β-cell functional impairment. This lends hope that the residual β-cell mass/function at T1D onset might be preserved and enhanced by therapeutic intervention. The differences in the remaining β-cell mass/function in T1D patients at diagnosis may also reflect individual variation in β-cell mass/function at birth and differences in the tempo of β-cell destruction which affect the rate of inter-individual disease progression [21,22]. Hence, better insight into the specific genes and mechanisms involved in β-cell destruction and functional impairment is of utmost importance to develop new treatment strategies to improve β-cell mass and/or function in newly-diagnosed T1D patients and to avoid β-cell impairment in risk individuals.

## 3. Expression of T1D Risk Genes in Islets

As of today, more than 50 T1D risk loci have been identified by GWAS and meta-analyses [7,23,24,25]. Together, these loci explain some 80%–85% of the heritability of T1D [26,27,28], which is noticeably higher than for other complex diseases [29]. Most of the T1D susceptibility loci harbor numerous genes, and for most of the loci the causal gene(s) and variant(s) await identification. If all genes located within 100 kilo bases from GWAS-significant SNPs are taken into consideration, the total number of genes in T1D loci is almost 1000 [5,30] all of which are potentially important causal candidate genes. Transcriptome profile studies have revealed that many of these genes are expressed in pancreatic islets and β-cells where they likely have a major impact on the triggering and development of T1D putatively by affecting the function and survival of the β-cells [2,3,31]. Such studies also identified clusters of genes regulated by cytokines and pinpointed key transcription factors. Further, studies analyzing protein–protein interactions between the products encoded by the genes in T1D loci have identified specific networks that are enriched in T1D candidate genes [2,31,32,33]. This suggests that a proportion of the genes located in T1D risk loci interact in networks at the islet level, further adding to the possibility that many genes affecting T1D risk do so by having functional roles in the islet cells. Importantly, it should be kept in mind that while transcriptome data supports that many candidate genes are expressed in islet tissue and β-cells, these same candidate genes may also be expressed in immune cells. In fact, it is highly likely that some of the causal genes in T1D affect disease susceptibility in cell-specific manners both at the islet and immune cell levels. In the sections below, we will, however, focus solely on candidate genes that have proven functional roles in β-cells.

## 4. T1D Candidate Genes that Affect Islet Inflammation and Apoptosis

**Figure 1:**
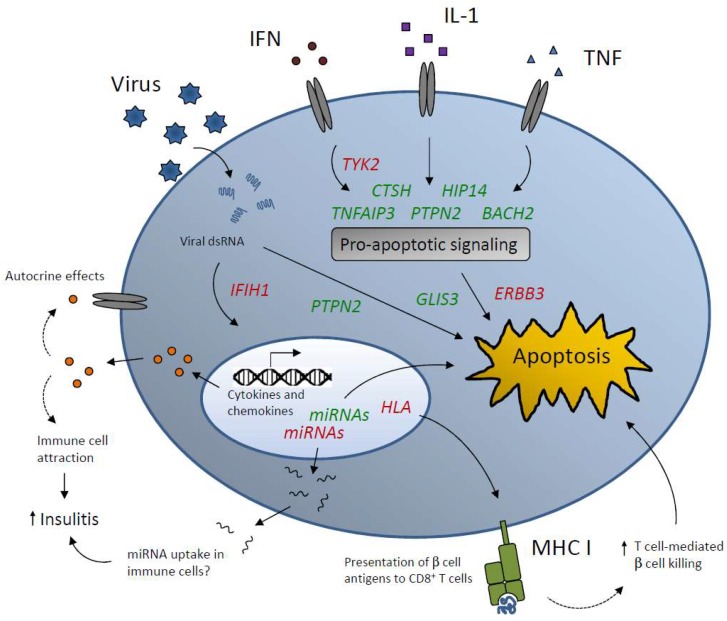
A number of type 1 diabetes (T1D) candidate genes are known to modulate β-cell apoptosis, viral infection, and islet inflammation. Genes marked in green and red designate protective and deleterious roles in the β-cell, respectively. Some of the T1D risk genes including *HIP14*, *TNFAIP3*, *TYK2*, and *CTSH* are regulators of cytokine effects at the level of pro-apoptotic signal transduction. Other genes such as *IFIH1* and *PTPN2* are involved in the cellular responses to viral double stranded RNA (dsRNA). Downstream signaling events include alterations in expression and secretion of inflammatory cytokines and chemokines that may exert detrimental autocrine effects and/or attract immune cells, thereby amplifying insulitis. Secreted micro RNAs (miRNAs) from β-cells may also increase islet inflammation and β-cell damage by modulating immune cell activity. Finally, genetic variants in *HLA* genes may cause β-cell hyperexpression of major histocompatibility complex (MHC) I presenting β-cell (neo)epitopes to cytotoxic T cells.

To prove causality of T1D candidate genes at the islet/β-cell level, functional investigations in relevant cellular model systems is necessary. Further, studies at the organism level such as genetically-modified mice with β-cell-specific gene knockout or overexpression are valuable for the understanding of how a given candidate gene affects the disease pathogenesis and contributes to disease risk. This field is still in its early stages, but a number of recent studies have identified candidate genes that affect β-cell function or survival in T1D settings (Figure 1). These studies along with the findings mentioned above, i.e., that many genes in T1D risk loci are expressed in islets and β-cells, substantiate that T1D is considered a consequence of both β-cell and immune function aberrations.

The *Huntingtin-interacting protein 14* (*HIP14*) gene encodes a palmitoyl transferase involved in the palmitoylation and trafficking of neuronal proteins [34]. However, in a search for novel T1D candidate genes utilizing a systems biology approach [35] on genome-wide linkage scan data, our group identified *HIP14* as a potential causal candidate gene in a T1D locus on Chr12 with function in β-cells [36]. Immunohistochemical staining of mouse pancreatic sections showed that HIP14 is nearly exclusively expressed in insulin-positive cells, suggesting β-cell-specific expression in islets. Knockdown experiments in insulin-secreting INS-1 cells revealed that HIP14 deficiency causes increased apoptotic cell death [36]. Conversely, increased levels of HIP14 protected against IL-1β-induced apoptosis, which correlated with reduced nuclear-factor kappa B (NFκB) activity. These effects of HIP14 are yet to be validated in primary β-cells and islets, but nevertheless, the findings obtained in INS-1 cells suggest that HIP14 has anti-apoptotic properties in β-cells. Interestingly, a recent study found that caspase 6 is palmitoylated and thereby inhibited by HIP14 in the mouse brain [37]. This may imply that the anti-apoptotic activity of HIP14 in β-cells is caused by decreased caspase 6 activity along with diminished NFκB signaling.

The *Erb-B2 Receptor Tyrosine Kinase 3 (ERBB3)* gene is located on Chr12q13.2 and is a member of the epidermal growth factor receptor family of tyrosine kinases. However, ERBB3 is unusual as it lacks intrinsic kinase activity, but mediates its effects via interaction with other receptors [38]. Several studies showed that SNPs (e.g., rs2292239) located in intron 7 in *ERBB3* are associated with T1D [7,25,39,40,41,42]. Furthermore, rs2292239 genotypes correlate with residual β-cell function and metabolic control during remission in newly-diagnosed children with T1D [43]. Evidence suggests that elevated expression of *ERBB3* plays an important role in the progression of several tumor forms [44] indicating that increased ERBB3 levels are linked to the regulation of cell proliferation and potentially apoptotic cell death. Indeed, a recent study by our group found that ERBB3 plays a role in cytokine-induced apoptosis in insulin-secreting cells. Noteworthy, cytokines suppress the expression of ERBB3 in both human islets and INS-1E cells, further suggesting that this gene is involved in the regulation of the detrimental effects of cytokines [43]. Thus, *ERBB3* is an interesting candidate gene that deserves further attention to clarify the mechanisms underlying the apoptosis-regulatory effects of the gene in β-cells.

Genetic variants in the *Interferon Induced Helicase C Domain 1 (IFIH1)* locus on Chr2q24.2 are associated with protection from T1D correlating with lower expression of the *IFIH1*-encoded protein product MDA5 (melanoma differentiation-associated protein 5), a cytoplasmic sensor of viral double stranded RNA (dsRNA) that activates a cascade of antiviral responses [45,46]. *IFIH1* is expressed in human islets and β-cells and is crucial for the immune response to enterovirus infection or exposure to synthetic dsRNA (polyinosinic:polycytidylic acid, poly(I:C)) [47,48]. Furthermore, the minor alleles of *IFIH1* associated with T1D have reduced activity against enterovirus infections [45]. Knockdown of *IFIH1* in INS-1E cells and primary β-cells had no impact on apoptosis induced by poly(I:C) or pro-inflammatory cytokines, but diminished the upregulation and release of specific cytokines and chemokines [47,49]. These findings suggest that one mechanism by which *IFIH1* contribute to β-cell destruction in T1D is by increasing the local production of inflammatory cytokines and chemokines, thereby exacerbating islet immune cell infiltration. Consistent with such a role of *IFIH1*, non-obese diabetic (NOD) mice with homozygous *Ifih1* knockout are fully protected from spontaneous diabetes, whereas NOD mice with heterozygous *Ifih1* knockout are partially protected [50]. Noteworthy, however, virus-induced acceleration of diabetes in NOD mice is prevented in heterozygous *Ifih1* NOD mice—an effect that correlates with a unique antiviral type I IFN signature and diminished insulitis with a shift towards a regulatory T cell response [50]. Based on these findings, attempts to reduce the expression or decrease the activity of *IFIH1*/*MDA5* may prove valuable as a future T1D intervention strategy.

SNPs in the *Tumor necrosis factor alpha-induced protein 3* (*TNFAIP3*) region on Chr6q23 are associated with T1D [7,51]. The protein product encoded by *TNFAIP3*, A20, is a cytoplasmic ubiquitin-editing protein that functions as a negative-feedback regulator of NF-κB signaling [52]. A20 is upregulated by cytokines (IL-1β and TNFα) in β-cells in an NFκB-dependent manner and in islets following syngeneic and allogeneic transplantation [53]. A20 possesses anti-apoptotic properties and A20 overexpression protects β-cells and islets from cytokine-induced apoptosis [53,54,55], affords protection in the early post-transplantation period [56], and rescues mice from chemically-induced diabetes [57]. The anti-apoptotic effect of A20 in β-cells is related to inhibition of NF-κB activation and nitric oxide production [55]. Recent evidence, however, also suggests that A20 inhibits pro-apoptotic cytokine signaling via c-Jun N-terminal kinase (JNK) and augments signaling via the Akt survival pathway [54], indicating that A20 has broad anti-apoptotic effects in β-cells. Interestingly, the T1D risk allele of a SNP (rs2327832) located upstream of *TNFAIP3* is associated with poorer residual β-cell function one year after T1D diagnosis [54] providing clinical evidence for a role of *TNFAIP3* in T1D development.

*GLIS Family Zinc Finger 3 (GLIS3)* on Chr9p24.2 is a candidate gene for both T1D and type 2 diabetes (T2D) [7,58]. *GLIS3* encodes a transcription factor, which is important for pancreas development and β-cell generation [59,60]. It is also required for maintaining mature β-cell function and mass [61]. GLIS3 regulates several key islet transcription factors as well as insulin gene transcription directly and via PDX1, MAFA, and NEUROD1 [60,61,62]. Knockdown experiments in human islet cells and INS-1E cells revealed that GLIS3-deficiency augments cytokine- and poly(I:C)-induced apoptosis by promoting the generation of a pro-apoptotic splice variant of BIM—a BH3-only protein belonging to the BCL2 family of apoptosis-regulatory proteins [63], suggesting an anti-apoptotic function of GLIS3. GLIS3 also upregulates *GLUT2* expression and enhances glucose-stimulated insulin secretion [61,63]. These effects correlate with the fact that variants in *GLIS3* are associated with fasting blood glucose and β-cell function in healthy individuals [64,65]. Remarkably, the SNPs associated with T1D and T2D, and related traits are in strong linkage disequilibrium (LD), suggesting that either a single genetic variant may be responsible for all reported disease associations [63] or that several variants in LD cooperatively contribute to disease susceptibility, e.g., by impacting gene expression [9]. As the SNPs are located in non-coding regions, it is conceivable that they increase diabetes susceptibility by lowering the *GLIS3* expression in the β-cells [63]. Forced expression of *GLIS3* in β-cells may therefore be promising as an anti-diabetic treatment strategy.

*PTPN2 (Protein Tyrosine Phosphatase, Non-Receptor Type 2)* on Chr18p11 is expressed in islets and β-cells and is upregulated in response to exposure to cytokines as well as poly(I:C) [47,66]. Functional studies showed that knockdown of *PTPN2* exacerbates apoptosis induced by IFN and poly(I:C) by increasing pro-apoptotic signaling via signal transducer and activator of transcription 1 (STAT1), JNK1, and BIM in INS-1E cells, primary β-cells and human islets [47,66,67]. These findings point towards a protective and anti-apoptotic role of *PTPN2* in β-cells. As the risk allele of an intronic T1D-associated SNP (rs1893217) in *PTPN2* causes decreased *PTPN2* expression, an attractive hypothesis is that this SNP confers disease susceptibility by sensitizing the β-cells to both immune- and virus-mediated apoptosis.

The T1D susceptibility locus on Chr15q25.1 contains *Cathepsin H (CTSH)*, which encodes a lysosomal cathepsin protease. Research by our group recently showed that *CTSH* expression is suppressed by cytokines in islets and β-cells [68]. Overexpression of *CTSH* in insulin-producing INS-1 cells afforded protection against cytokine-induced apoptosis associated with decreased signaling via the JNK and p38 pathways and reduced expression of the pro-apoptotic factors c-Myc, Bim and DP5, suggesting an anti-apoptotic function of CTSH in β cells [68]. CTSH is also a positive regulator of insulin transcription [68]. The T1D-associated SNP rs3825932 located in intron 1 of *CTSH* affects the expression level of *CTSH* in a genotype-dependent manner in multiple tissues [69] . Interestingly, we found that rs3825932 variants are critical determinants of β-cell function in children with recent-onset T1D and in healthy adults [68]. Moreover, carriers of the variant causing reduced *CTSH* expression have poorer β-cell function and a faster disease progression compared to carriers of the variant causing high *CTSH* expression [68]. Ongoing activities in our group are aiming to further investigate the role of *CTSH* in T1D and the underlying molecular mechanisms.

*Tyrosine Kinase 2 (TYK2)* is located on 19p13.2 and harbors a non-synonymous SNP that causes a missense mutation in *TYK2,* which is associated with a lower risk of T1D [70]. *TYK2* encodes a tyrosine kinase that interacts with the cytoplasmic domain of IFN receptors and is thus involved in transmitting IFN signaling. Mice deficient in *Tyk2* or mice with mutated *Tyk2* causing lower Tyk2 expression are more sensitive to virus-induced diabetes and display increased virus titers and IFNα levels compared to wildtype mice, suggesting that low expression of Tyk2 correlates with a less efficient antiviral response in mice [71]. This model probably reflects a specific subtype of acute virus-induced T1D as knockdown of *TYK2* in insulin-secreting human EndoC-βH1 cells and islets leads to decreased poly(I:C)-induced STAT1/2 activation, reduced production of IFNα and CXCL10, lower major histocompatibility complex (MHC) I expression, and diminished apoptosis [72]. These observations support that TYK2 is likely to play a critical role for antiviral mechanisms and islet inflammatory processes in T1D. The exact role of TYK2 and how the expression level of TYK2 may modulate T1D risk, however, awaits clarification. It is conceivable that TYK2 might have opposing roles depending on the tissue, i.e., islets vs. immune cells, and modify T1D risk in a stimulus-dependent manner, i.e., whether disease is triggered by e.g., viral infection or other environmental/genetic triggers.

*BACH2 (BTB Domain and CNC Homolog 2)* on Chr6q15 encodes a transcription factor of the Basic Region-Leucine Zipper family and is involved in both innate and adaptive immunity [73]. It is expressed in β-cells and upregulated by pro-inflammatory cytokines [74]. Knockdown of *BACH2* exacerbates pro-inflammatory cytokine-induced apoptosis in EndoC-βH1 cells, human islets and primary rat β-cells by potentiating pro-apoptotic signaling via JNK1 and BIM, indicating an anti-apoptotic function of BACH2 in β-cells [74]. Interestingly, silencing *BACH2* expression also causes decreased *PTPN2* expression in response to cytokine treatment, suggesting functional interactions between these two T1D candidate genes at the level of the β-cell [74].

## 5. Genetic Risk-Score of Islet-Expressed T1D Candidate Genes as Predictor of Disease Progression

As evident from above, a number of candidate genes for T1D play crucial roles in β-cells by affecting their vulnerability to undergo apoptosis, viral infection, and/or cytokine/chemokine production. It is highly likely that these genes act in concert to affect disease risk as well as disease progression once clinically manifested.

We recently hypothesized that a genetic risk-score (GRS) of islet-expressed T1D candidate genes could be a valuable method to predict disease progression in newly-diagnosed patients, the rationale being that islet-expressed candidate genes would be the best predictors of β-cell function. We therefore investigated the impact of an increasing GRS on β-cell function and glycemic control during the first year after T1D onset [75]. The GRS was constructed from 11 T1D risk genes, including some of the genes described in detail above that we found to be expressed and cytokine regulated in human islets. For each additional risk allele, i.e., for each unit increase in the GRS, a decreased β-cell function and a worsened glycemic control from 6 to 12 months after disease onset were observed [75]. Remarkably, we also found that several of the 11 genes used in the GRS interacted in a common network, further supporting that they cooperate to regulate T1D risk at the β-cell level. Hence, these results indicate that profiling of selected genetic variants serving as markers of β-cell risk genes holds the potential to better predict disease progression in recent-onset patients and whether T1D advance in risk individuals.

## 6. MHC and the β-Cell

MHC, which encodes the human leukocyte antigens (HLA), accounts for approximately half of the genetic risk to T1D. Traditionally, the role of MHC in T1D etiology is considered to be in central immunotolerance and antigen presentation. However, evidence supports that the β cell itself may be an active player in the MHC-conferred susceptibility. Hence, β-cell hyperexpression of MHC class I, including β2-microglobulin, is consistently seen in pancreatic samples from human T1D patients [76,77,78] and following cytokine exposure of human islets [3]. β-cell-induced immune cell activation via MHC I-mediated interaction is likely an important mechanism by which the β cell is recognized and killed by the immune system (Figure 1).

We have previously proposed that part of the genetic component of T1D is caused by variation in genes regulating posttranslational modification (PTM) processes in the β-cell [79]. We identified a number of genes involved in generation of various PTMs that all have T1D-associated SNPs mapping to either introns or up- or downstream regions (within 5 kb of the gene). This provides support that such genes contribute to the generation of PTMs of β-cell proteins that could lead to peptide neo-epitopes with antigenic potential when presented by T1D-associated HLA class I and II molecules.

## 7. Non-Coding RNAs Regulating the β-Cell Genome

Several classes of non-coding RNAs have been investigated in relation to diabetes, however, the best studied class is microRNAs (miRNAs), which represent a class of evolutionary conserved 22–25 nucleotides long molecules that function as posttranscriptional regulators by binding to the 3′ untranslated region (3′UTR) of target mRNAs, causing translational repression or mRNA degradation [80]. Evidence suggests that miRNAs are critical for pancreatic development and differentiation, and for β-cell function [81,82]. The expression of multiple miRNAs is modulated by pro-inflammatory cytokines or glucose in islets and β-cells. These miRNAs seem to modulate insulin expression/secretion, proliferation, and/or apoptosis [83].

The T1D susceptibility loci harbor several miRNA genes [5]. T1D risk SNPs may affect T1D susceptibility by altering the seed-sequence in miRNAs or by changing the miRNA target sites in protein-coding genes. Alternatively, they may cause alterations in the structure of flanking regions, thereby modulating the accessibility for miRNA binding. Accordingly, T1D SNPs have been shown to disrupt or introduce miRNA binding sites in coding candidate genes [84]. Variation in miRNA genes and miRNA binding sites in target genes have also been associated with T2D [85].

Recently, a study reported that β-cells secrete exosomal miRNAs which can be taken up by neighbouring β cells [86]. This opens up for the fascinating possibility that miRNA-mediated communication between the β-cells, between β-cells and other islet cell types, and/or between β-cells and immune cells, may play an important pathogenic role. Only a few studies have addressed the role of miRNAs specifically in relation to β-cell function in human T1D. Recently, Samandari et al. [87] identified a miRNA signature that correlated with residual β-cell function up to one year after T1D diagnosis in children and adolescents, and Grieco et al. [88] identified miRNAs that regulate the expression of pro-apoptotic proteins in human β-cells—thus regulating apoptotic cell death. Intriguingly, there was no overlap in identified miRNAs between these studies, indicating that the pathway/network regulatory effects of miRNAs are both tissue and context specific.

## 8. Conclusions

T1D risk is conferred by genetic aberrations of both the immune system and the β-cell. Characterization of the β-cell transcriptome and the molecular disease mechanisms taking place in the β-cells in T1D are crucial to understand the disease and for developing new strategies to prevent β-cell destruction in T1D. New integrative approaches that combine different omics-technologies and functional experiments are in great need and should further our understanding of the molecular disease mechanisms in the β-cell.

The disease is complex. It is also heterogeneous. Such heterogeneity might be explained by a combination of individual risk profiles and exposure to different environmental factors that can be very well reflected at the level of the β-cell. In some individuals, an excessive innate immune response might prevail, while in others, the β-cells may be extraordinarily vulnerable to immune-mediated apoptosis. This suggests that different therapeutic strategies will be required depending on the genetic background of the affected individuals. The challenge will be to decipher T1D complexities and move towards precision medicine approaches.
